# Supplementary material for: Comparative analysis of salivary antimicrobial resistance genes in dental students: A PCR and questionnaire study
Source: PLoS One. 2025 Jan 16;20(1):e0315450. doi: 10.1371/journal.pone.0315450 (PMC11737694; doi:10.1371/journal.pone.0315450)
Supplement: Attachment — Submitted filename: review.docx [file pone.0315450.s001.docx]

The manuscript by Marwan Mansoor Mohammed et al., examining the prevalence of antimicrobial resistance (AMR) genes in the saliva of dental students, aims to fill a gap in the literature regarding AMR in the UAE by utilizing PCR techniques and questionnaire data. Despite its relevant topic, the study struggles with significant issues, including methodological weaknesses, poor data interpretation, and a lack of innovation, which undermine its scientific credibility and suitability for publication in PLOS ONE. The absence of a clear rationale for gene selection, inadequate statistical analysis, superficial interpretation of results, and insufficient integration into the wider AMR discourse highlight the urgent need for extensive revisions to elevate the manuscript to the standards expected by reputable scientific journals.

Firstly, the study fails to provide a rationale for selecting specific AMR genes for analysis. The absence of this critical justification undermines the scientific foundation of the research, casting doubts on the relevance and scope of the study. This omission is particularly problematic as it hinders the reader’s ability to fully understand the intentions and potential impact of the research within the broader field of AMR studies. By addressing this issue, the study could potentially make a significant contribution to the field of AMR research in the UAE.

Moreover, the manuscript's results section needs robust statistical analysis, omitting essential details such as the statistical tests, p-values, or confidence intervals. This lack of rigorous statistical documentation is a severe shortfall, rendering the findings unreliable and their conclusions untrustworthy. This oversight must meet the stringent standards for a journal committed to rigorous scientific scrutiny.

The manuscript also presents a wealth of data on the distribution of various AMR genes among dental students but does so without adequate analysis of how these data correlate with other variables like health status or antibiotic use history. The absence of in-depth analytical insight into these potential relationships means the research does not fully utilize its data to advance understanding of AMR dynamics, which is necessary for impactful research. A more comprehensive analysis is needed to fully understand the implications of the data.

Additionally, discussing findings related to different resistance genes needs more contextualization within the broader AMR research landscape. The data are presented in isolation, without comparative analysis to existing literature, diminishing the manuscript’s scientific value. By not linking its findings to the wider discourse on antimicrobial resistance, the study misses an opportunity to make a meaningful contribution to the field.

The manuscript frequently notes the high prevalence of various ARGs, suggesting their potential for dissemination within the oral microbiome. However, it needs to contextualize these findings within global research trends or epidemiological data. This lack of a nuanced discussion that critically examines how the results compare with global trends in AMR prevalence, especially given the unique context of the UAE, further limits the manuscript’s impact.

Functional assays or additional validation do not support the interpretation of findings, particularly the speculative conclusions drawn from the PCR data regarding the hydrolytic activities of enzymes like the *bla*_CTX-M_ variants. This overinterpretation relative to the methodological rigor presented leads to conclusions not substantiated by the data. The speculative nature of these interpretations, especially the reliance on Ct values to indicate gene concentration and their clinical relevance, is scientifically unsound.
